# Priming Mesenchymal Stem Cells with Endothelial Growth Medium Boosts Stem Cell Therapy for Systemic Arterial Hypertension

**DOI:** 10.1155/2015/685383

**Published:** 2015-08-02

**Authors:** Lucas Felipe de Oliveira, Thalles Ramos Almeida, Marcus Paulo Ribeiro Machado, Marilia Beatriz Cuba, Angélica Cristina Alves, Marcos Vinícius da Silva, Virmondes Rodrigues Júnior, Valdo José Dias da Silva

**Affiliations:** ^1^Physiology Division, Natural and Biological Sciences Institute, Triangulo Mineiro Federal University, 38025-015 Uberaba, MG, Brazil; ^2^Immunology Division, Natural and Biological Sciences Institute, Triangulo Mineiro Federal University, 38025-015 Uberaba, MG, Brazil

## Abstract

Systemic arterial hypertension (SAH), a clinical syndrome characterized by persistent elevation of arterial pressure, is often associated with abnormalities such as microvascular rarefaction, defective angiogenesis, and endothelial dysfunction. Mesenchymal stem cells (MSCs), which normally induce angiogenesis and improve endothelial function, are defective in SAH. The central aim of this study was to evaluate whether priming of MSCs with endothelial growth medium (EGM-2) increases their therapeutic effects in spontaneously hypertensive rats (SHRs). Adult female SHRs were administered an intraperitoneal injection of vehicle solution (*n* = 10), MSCs cultured in conventional medium (DMEM plus 10% FBS, *n* = 11), or MSCs cultured in conventional medium followed by 72 hours in EGM-2 (pMSC, *n* = 10). Priming of the MSCs reduced the basal cell death rate *in vitro*. The administration of pMSCs significantly induced a prolonged reduction (10 days) in arterial pressure, a decrease in cardiac hypertrophy, an improvement in endothelium-dependent vasodilation response to acetylcholine, and an increase in skeletal muscle microvascular density compared to the vehicle and MSC groups. The transplanted cells were rarely found in the hearts and kidneys. Taken together, our findings indicate that priming of MSCs boosts stem cell therapy for the treatment of SAH.

## 1. Introduction

Systemic arterial hypertension (SAH) is a clinical syndrome characterized by systolic blood pressure levels close to or greater than 140 mmHg or diastolic blood pressure close to or greater than 90 mmHg, affecting approximately 15–20% of the worldwide population [1]. Great advances have been achieved related to the understanding of the pathogenesis of SAH, and there are many available medications for maintaining blood pressure at the desirable levels. However, there are many unanswered questions about the pathophysiological basis of this syndrome, and we do not have a definitive treatment for hypertensive patients, especially patients with resistant hypertension. Therefore, despite the marked advances over the last few decades, SAH still remains as a huge public health problem that deserves intensive research to develop new and more effective therapies.

To achieve this, the experimental model of spontaneous hypertension in rats (spontaneously hypertensive rats—SHRs) is extensively utilized in research labs because it is an excellent model to study essential SAH; in addition, its pathogenesis is very similar to human SAH. Key alterations in SHRs include sympathetic hyperactivity [2], microvascular rarefaction [3], and endothelial dysfunction [4, 5].

Mesenchymal stem cells (MSCs), also called bone marrow stromal cells or multipotent mesenchymal stromal cells, have the property of plastic adherence under standard culture conditions, have the capacity of* in vitro* differentiation into osteoblasts, chondroblasts, and adipocytes, and express a typical phenotype profile [6, 7]. MSCs have an important neoangiogenic action, which is confirmed by studies showing an elevated capacity of these cells to secret many bioactive factors involved in new vessel formation, such as vascular endothelial growth factor (VEGF), hepatocyte growth factor (HGF), fibroblast growth factor-2 (FGF-2), angiopoietin-1 (Ang-1), monocyte chemoattractant protein-1 (MCP-1), interleukin-6 (IL-6), placental growth factor (PLGF), and protein Cyr61 (cysteine-rich, angiogenic inducer 61), among others [8–10]. Additionally, it is believed that pericytes represent the MSCs, which are localized in the whole organism associated with blood vessels [11]. Pericytes and vascular endothelial cells exhibit an interdependent relationship, wherein soluble factors and physical interactions synergistically contribute to blood vessel structure, both for their formation and for their maintenance [12]. Therefore, it is possible that MSCs exert modulatory action on endothelial function. These ideas reinforce a possible role of MSCs in SAH pathophysiology, especially acting on peripheral vascular resistance, both interfering with new microvessel formation and/or modulating endothelial function.

Taking into account that the endothelial dysfunction and microvascular rarefaction are important alterations in the hypertensive state [4, 5], the improvement of these parameters can produce therapeutic benefits related to arterial hypertension. Moreover, considering the therapeutic potential of stem/progenitor cells from bone marrow to improve vascular rarefaction and/or endothelium dysfunction in SHR, our hypothesis is that the priming of MSCs with endothelial basal medium plus growth factors (endothelial growth medium—EGM-2), which appears to potentiate their stemness, angiogenic capability, and therapeutic potential, could yield a safe and efficient therapeutic alternative to arterial hypertension.

## 2. Materials and Methods

### 2.1. Animal Selection

All animals were obtained from the animal facility of the Natural and Biological Sciences Institute of Federal University of Triangulo Mineiro. The animals were maintained under stable conditions with free access to water and food. All of the experimental proceedings employed in this study complied with the* Guide for the Care and Use of Laboratory Animals* published by The US National Institutes of Health (NIH publication number 85-23, revised 1996).

In the 5 days before treatment, the animals were subjected to indirect arterial pressure recording using the tail artery occlusion method and an indirect pressure monitor, LE5000 model (Letica Scientific Instruments, Barcelona, Spain), which allows the indirect measurement of systolic arterial pressure. In this study, we used only female SHRs whose systolic arterial pressure (SAP) was higher than 160 mmHg.

### 2.2. Mesenchymal Stem Cell Isolation

MSCs were obtained from the bone marrow of male SHRs as described previously [13]. Briefly, the bone marrow was obtained from femurs, tibias, and humeri and centrifugation, and additional differential centrifugation using Ficoll-Paque at 400 g for 40 minutes was performed; then, the material was resuspended in conventional medium consisting of Dulbecco's modified Eagle medium, DMEM (Invitrogen), and 10% fetal bovine serum, FBS (Gibco), and supplemented with 100 U/mL penicillin G and 100 *μ*g/mL streptomycin (Invitrogen) and then plated into T75 flasks. After 72 hours, adherent cells were detached with 0.25% trypsin/ethylenediamine tetraacetic acid (Invitrogen) and replated at 20,000 cells/cm^2^. The cells obtained in the extraction were considered P0, and, at each new replating, the cells advanced one passage. In the present study, we used MSCs between the fourth and seventh passages for all procedures. During the 72 hours before each experimental protocol, the cells were cultivated on conventional medium or endothelial growth medium (EGM-2, Lonza, USA) plus 10% of fetal bovine serum, FBS (Gibco), and supplemented with 100 U/mL penicillin G and 100 *μ*g/mL streptomycin (Invitrogen), hydrocortisone, human basic fibroblast growth factor (hFGFb), vascular endothelial growth factor (VEGF), insulin-like growth factor-1 (IGF-1), and ascorbic acid and heparin (Lonza, USA).

### 2.3. MSCs Immunophenotypic Characterization

The immunophenotypic characterization of MSCs (at the fourth to seventh passages) before priming was accomplished using flow cytometry with monoclonal antibodies to the markers, CD11b, CD29, CD31, CD34, CD45, and CD117(cKit) (BD Biosciences Inc., San Jose, CA; eBiosciences, San Diego, CA, USA), conjugated to phycoerythrin (PE), which reacts with homologue rats antigens. Cell phenotype analysis was performed on 20,000 events for each sample, using a FACSCalibur flow cytometer with CELLQUEST software (Becton, Dickinson and Company, San Jose, CA).

### 2.4. Cellular Differentiation

MSCs in the culture were stimulated at the fourth to seventh passages, using a specific protocol to differentiate them into osteoblasts and adipocytes. The cell differentiation protocol was based on Neuhuber et al. [13].

For the induction of osteogenic differentiation, MSCs were cultured in osteogenic induction medium consisting of DMEM plus 15% FBS, 1% penicillin/streptomycin, 100 nM dexamethasone, 50 *μ*M ascorbate-2-phosphate, and 10 mM glycerol-phosphate. The osteogenic induction medium was replaced every 3-4 days, and, on the eighteenth day, the analysis of differentiation was performed using Alizarin Red dye (Sigma Aldrich, USA), which stains bone matrix red.

To induce adipogenic differentiation, MSCs were cultured in adipogenic induction medium, consisting of DMEM plus 15% FBS, 1% penicillin/streptomycin, 1 *μ*M dexamethasone, 0.5 mM isobutylmethylxanthine, 10 *μ*g/mL insulin, and 100 *μ*M indomethacin. After 3 days in adipogenic induction medium, the medium was replaced by adipogenic maintenance medium for 24 hours, consisting of DMEM plus 15% FBS, 1% penicillin/streptomycin, and 10 *μ*g/mL insulin. After 24 hours, the adipogenic maintenance medium was again replaced by adipogenic induction medium for 3 days, when it was replaced by adipogenic maintenance medium for further 24 hours. After completing 3 cycles of media changes, the cells were maintained for 5 days in the maintenance medium, when the analysis of differentiation was performed using Oil Red dye (Sigma Aldrich, USA), which stains lipids orange.

### 2.5. Cell Death

Cell death evaluation was performed through flow cytometry using an FITC Annexin V Apoptosis Detection Kit II (BD Pharmingen) according to the manufacturer's instructions. Briefly, MSCs were plated at a density of 25,000 cells/cm^2^, and, after 72 hours of cultivation in conventional medium or EGM-2, the cells were detached, suspended in annexin V buffer, and incubated with FITC-annexin V and propidium iodide for flow cytometry reading. Following the kit instructions, the* in vitro* basal cell death rate was determined as the percentage of cells that were positively stained with both dyes.

### 2.6. Cell Transplantation

The MSCs were cultured in conventional medium and, 72 hours before cell transplantation, the cells were maintained in conventional medium or EGM-2 medium. After 72 h, the cells were detached from the flasks and cell viability was assessed by means of Trypan Blue exclusion test. Only cultures with cell viability higher than 95% were used for transplantation. In previous cell injection, the cells were stained with CM-DiI cell tracker (Molecular Probes, USA) and then intraperitoneally injected at a concentration of 5 × 10^6^ viable cells/animal. The experimental groups were formed by animals that received MSCs cultured in conventional medium (MSC, *n* = 11), animals that received MSCs primed with EGM-2 medium (pMSC, *n* = 10), and the control animals that received 1 mL of the vehicle (saline, *n* = 11).

### 2.7. Experimental Protocol

Systolic arterial pressure (SAP) was monitored by means of tail artery occlusion method 5 days before and during the 10 days after treatment. At the end of this observation period, for direct recordings of hemodynamic parameters, all animals were anesthetized with tribromoethanol (250 mg/kg, i.p.) and had their right femoral artery catheterized. After 24–48 hours of surgical recovery, arterial blood pressure was directly recorded over 60 minutes using an Analog to Digital Converter (DI-720USB, Dataq Instruments, Inc., Akron, OH, USA) and data acquisition software (Windaq Pro+, Dataq Instruments, Inc., Akron, OH, USA) at a sampling rate of 1 KHz. At the end of this recording period, after a new anesthesia session (sodium thiopental, 40 mg/kg, i.p.), a catheter was inserted into the left common carotid artery for injection of acetylcholine (3–25 *η*g/kg) or sodium nitroprusside (0.5–4 *μ*g/kg) to indirectly evaluate the systemic endothelial function based on depressor responses to endothelial-dependent and endothelial-independent vasodilators, respectively. At the end of the experimental protocol, the heart weight was measured, and kidney, heart, and skeletal muscle were collected.

### 2.8. Functional Analysis

Mean values of SAP, mean arterial pressure (MAP), diastolic arterial pressure (DAP), and pulse interval (PI) were calculated for each 60-minute period of recording. For the cardiovascular variability study, the signals of arterial pressure (AP) were processed using software (PRE24 software, kindly provided by Dr. Alberto Porta, University of Milan, Italy) to generate beat-to-beat time series of PI, DAP, and SAP. The variance of these values in each period was considered a variability index in the time domain.

The variability of PI, DAP, and SAP was also evaluated in the frequency domain using an autoregressive spectral analysis method. The theoretical and analytical proceedings are described in previous studies [14, 15]. Briefly, beat by beat time series of PI, DAP, and SAP were divided into serial segments of 200 beats, wherein all successive segments were overlapped by 50% (100 beats) on the previous segments (Welch's method). Using stationary time series segments, autoregressive parameters were estimated using Levinson-Durbin's method, and the model order was chosen according to Akaike's criteria [14–16]. Then, on each individual stationary segment of 200 beats, spectral decomposition was performed using appropriate software (LA24 software, kindly provided by Dr. Alberto Porta, University of Milan, Italy). The normalization procedure, applied only to the variability of the PI, was performed by dividing the power of the low frequency component (low frequency—LF, 0.20–0.80 Hz) or high frequency (high frequency—HF, 0.80–3 Hz) by total spectral power, which is subtracted from the power of the very low frequency band (very low frequency—VLF, 0.01 to 0.20 Hz), and multiplying the result by 100 [14, 15]. The spectral parameters obtained for each individual stationary segment of 200 beats were mediated, and the average values resulting from 60 minutes of recording were calculated for each animal.

For the endothelial function test, the arterial pressure responses to different doses of acetylcholine and sodium nitroprusside were evaluated based on the percentage decrease in MAP after the administration of each dose, using Acqknowledge Software, version 3.5.7 (Biopac Systems, USA).

### 2.9. Microvascular Density

To study the capillary density in the skeletal muscle, immunohistochemical labeling using (5-Bromo-4-Chloro-3-Indolyl Phosphate/NitroBlue Tetrazolium) BCIP/NBT Liquid Substrate System (Sigma Aldrich, USA) was performed. With this method, a substrate is converted by alkaline phosphatase present in endothelial cells to produce a dark blue stain. Briefly, previously frozen gastrocnemius muscle fragments were sectioned in a cryostat and allowed to react with BCIP/NBT substrate for 10 minutes at room temperature and contrastained with eosin dye. Using an inverted microscope (Axio Observer Z1, Carl Zeiss, Germany), twenty random fields were acquired, and the analysis was performed by counting the number of vessels stained in blue and the number of entire cells in each field. Microvascular density was determined as the ratio between the vessels and entire cell numbers.

### 2.10. Detection of Transplanted Cells

Previously frozen hearts and kidney fragments were sectioned in a cryostat and stained with DAPI (Sigma Aldrich, USA) for nuclei staining. The tissues were analyzed using a laser confocal microscope (LSM 710, Carl Zeiss, Germany) to locate the cells labeled with CM-DiI cell tracker stained in red.

### 2.11. Statistical Analysis

All parameters were expressed as the mean (±sem). Normality (Kolmogorov-Smirnov) and variance homogeneity (Bartlett) were determined for all data. For the parametric data, differences among the groups were analyzed using one-way ANOVA or two-way ANOVA for repeated measurements followed by Tukey's multiple comparisons test. For nonparametric data, the differences among the groups were analyzed using Kruskall-Wallis ANOVA or Mann-Whitney tests when appropriate. Values of *p* < 0.05 were considered statistically significant.

## 3. Results

### 3.1. Characterization of MSCs

**Figure 1:**
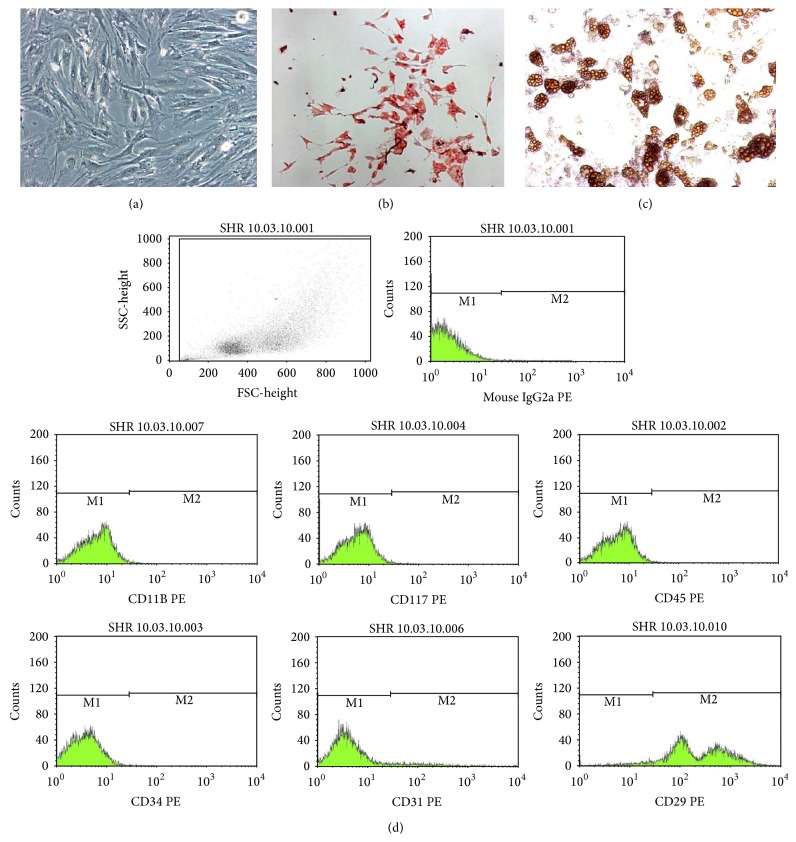
Phase-contrast micrographs of MSCs in culture (Panel (a)), showing the typical fibroblastoid morphology. Differentiation of MSCs, which were cultured in osteogenic and adipogenic medium as previously described. Calcium deposited in the extracellular matrix is stained red by Alizarin Red (Panel (b)). Lipid vacuoles are stained orange with Oil Red O (Panel (c)). Magnifications, ×100 (Panels (a)–(c)). (Panel (d)) Immunophenotypic profile of MSCs cultured in conventional medium. Flow cytometry expression of selected molecules (CD11b, CD117(cKit), CD45, CD34, CD31 and CD29) by MSC population.

Characterization of the stem cells investigated in the present study was performed as indicated by the International Society for Stem Cell Therapy and the Mesenchymal and Tissue Stem Cell Committee [7]. The MSCs exhibited all of their defining characteristics, such as plastic adherence capability, fibroblastoid format (Figure 1(a)), and* in vitro* osteogenic and adipogenic differentiation using specific differentiation medium demonstrating their multipotential properties (Figures 1(b) and 1(c)). As expected, MSCs presented the immunophenotype consistent with the accepted definition, namely, CD45^−^, CD29^+^, CD117^−^, CD31^−^, CD11b^−^, and CD34^−^; then, cell culture contamination with hematopoietic stem cells, leukocytes, endothelial cells, and macrophages was eliminated (Figure 1(d)).

### 3.2. Cell Death

**Figure 2:**
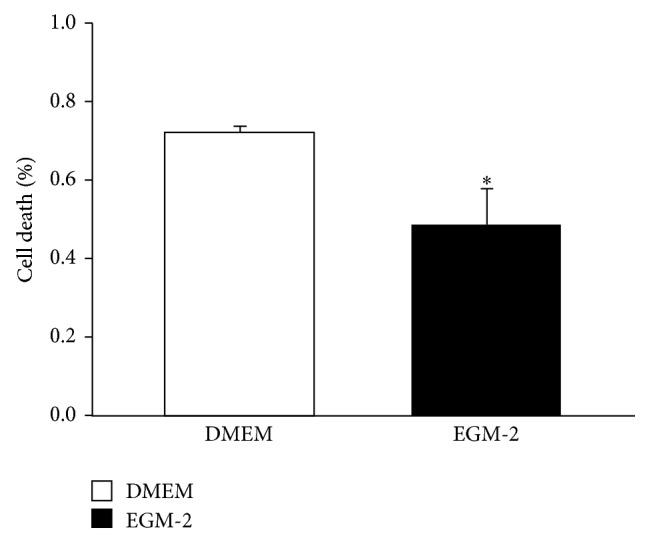
Cell Death. Percentage of dead cells that are positive for both Anexin V and propidium iodide in the population of cells cultured on conventional medium (DMEM) or stimulated with EGM-2 (EGM-2). Values indicated as the mean ± sem. ^*∗*^ *p* < 0.05 vs DMEM group. (*n* = 5 for both groups).

The cell death study showed a significant reduction of the* in vitro* basal cell death rate and, consequently, an increase in the percentage of viable cells on MSCs cultured for 72 hours in EGM-2 compared with the cells cultured in conventional medium (Figure 2).

### 3.3. Measurement of Arterial Blood Pressure and Heart Rate

**Figure 3:**
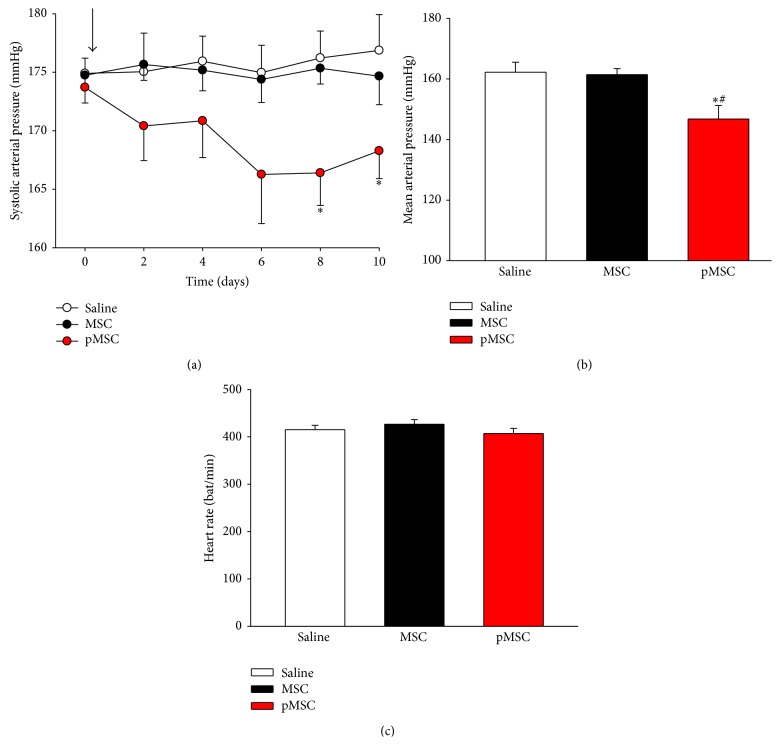
(a) Time course of systolic arterial pressure (SBP). Indirect measurement of systolic blood pressure from day 0 until day 10 after treatment. Arrow indicates the time of transplantation. (b) Values of mean arterial pressure obtained at the end of the observation period through direct measurement of arterial blood pressure. (c) Values of heart rate of three experimental groups measured at the end of the observation period. Values indicated as the mean ± sem. ^*∗*^ *p* < 0.05 versus saline; ^#^ *p* < 0.05 versus MSC.

Basal values of systolic arterial pressure before treatment did not differ among the groups (174.90 ± 1.3 mmHg for the saline group; 174.74 ± 0.88 mmHg for the MSC group; and 173.70 ± 1.33 mmHg for the pMSC group). However, the time course follow-up of indirect SAP measurements has shown a prolonged reduction (10 days) of tensional levels (approximately 10–15 mmHg) after i.p. administration of MSCs cultured for 72 hours in EGM-2 (Figure 3(a)). Similarly, as shown in Figure 3(b), the transplantation of pMSCs produced a significant reduction in directly recorded mean arterial blood pressure levels compared to vehicle or MSCs cultured in DMEM (146.77 ± 14.15 mmHg versus 162.22 ± 10.29 mmHg and 161.43 ± 6.63 mmHg, resp., *p* < 0.05). The treatments did not induce alterations in heart rate (HR) in the animals because there were no differences among the three experimental groups (Figure 3(c)).

### 3.4. AP and HR Variability

The treatment with MSCs cultured in conventional medium and those cultured in EGM-2 modified the variance of DAP, which was different compared to the saline group. The DAP spectral components of low frequency (LF) in the MSC group were statistically lower than those in the saline group (Table 1).

The analysis of HR variability showed differences in LF and LF (nu) components in the MSC group, which were statistically lower than those in the saline group. All the other data did not show any alterations (Table 2).

### 3.5. Endothelial Function Tests

**Figure 4:**
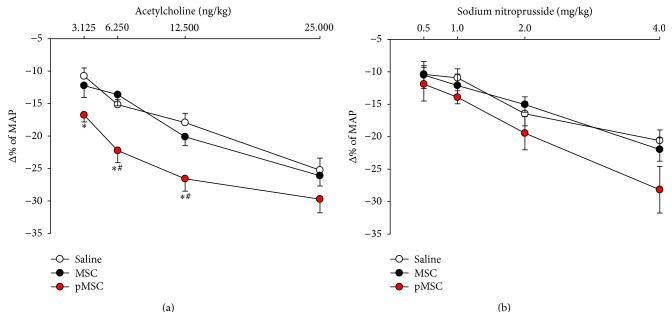
Study of endothelial function. Improvement in endothelial function was analyzed based on the percentage reduction of MAP in response to injections of different doses of acetylcholine (a). Injections of sodium nitroprusside were performed to control the endothelium-independent vasodilator response (b). Values indicated as the mean ± sem. ^*∗*^ *p* < 0.05 versus saline; ^#^ *p* < 0.05 versus MSC.

The change in blood pressure levels in response to different doses of vasorelaxant drugs acting on the vascular endothelium (acetylcholine) or vascular smooth muscle (sodium nitroprusside) allows the study of endothelial function. As shown in Figure 4, endothelial function was evaluated based on the relative decrease in arterial blood pressure in response to distinct doses of acetylcholine, and our findings showed an improvement in vasodilation response in animals treated with MSCs primed with EGM-2 medium compared to vehicle or MSCs cultured in DMEM for injections of Ach 6.25 ng/kg (22.24 ± 5.26% versus 15.17 ± 2.02% and 13.63 ± 4.84%, resp., *p* < 0.05) and 12.5 ng/kg (26.58 ± 5.42% versus 17.93 ± 3.79% and 20.12 ± 3.85%, resp., *p* < 0.05). The administration of the Ach 3.125 ng/kg dosage produced an improvement in the vasodilation response in the animals treated with pMSC compared to vehicle, but not to MSCs cultured in DMEM (16.76 ± 2.81% versus 10.76 ± 3.30%, *p* < 0.05; 16.76 ± 2.81% versus 12.23 ± 5.15%, *p* = 0.09). The Ach 25 ng/kg dosage also produced an improvement in the vasodilation response in the pMSC group, although it was not statistically significant compared with the saline or MSC group (29.71 ± 5.96 versus 25.26 ± 4.95% and 26.11 ± 4.47%, resp., *p* = 0.226). There were no differences between groups for all doses of sodium nitroprusside, which represents a vasodilation response that was independent of the endothelium.

### 3.6. Cardiac Relative Weight

**Figure 5:**
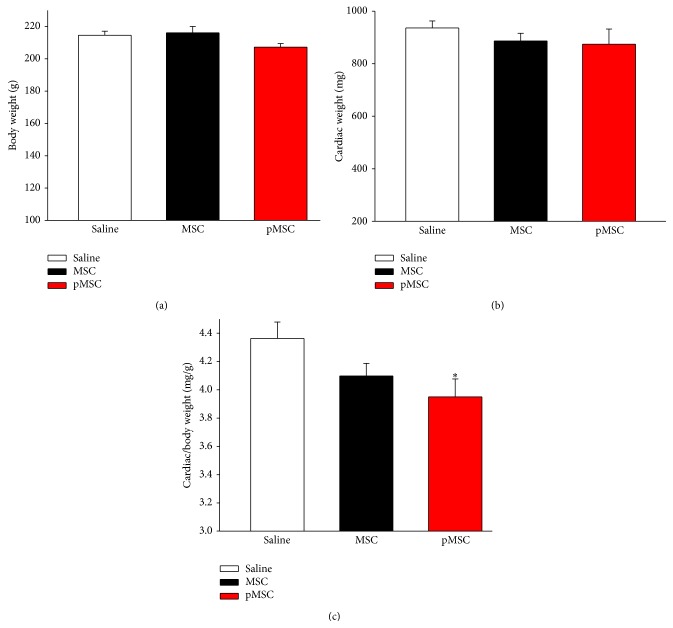
Analysis of cardiac hypertrophy. (a) Values of body weight (g), (b) cardiac weight (mg), and (c) cardiac/body weight ratio (mg/g). Values indicated as the mean ± sem. ^*∗*^ *p* < 0.05 versus saline.

Consistent with arterial blood pressure reduction, treatment with MSCs primed with EGM-2 reduced cardiac hypertrophy, based on the cardiac weight to body weight ratio, compared with saline (3.95 ± 0.35 versus 4.36 ± 0.38, *p* < 0.05), but not compared with the MSC group (3.95 ± 0.35 versus 4.09 ± 0.26, *p* = nonsignificant). There were no differences in body weight and absolute heart weight among the three groups (Figure 5).

### 3.7. Microvascular Density

**Figure 6:**
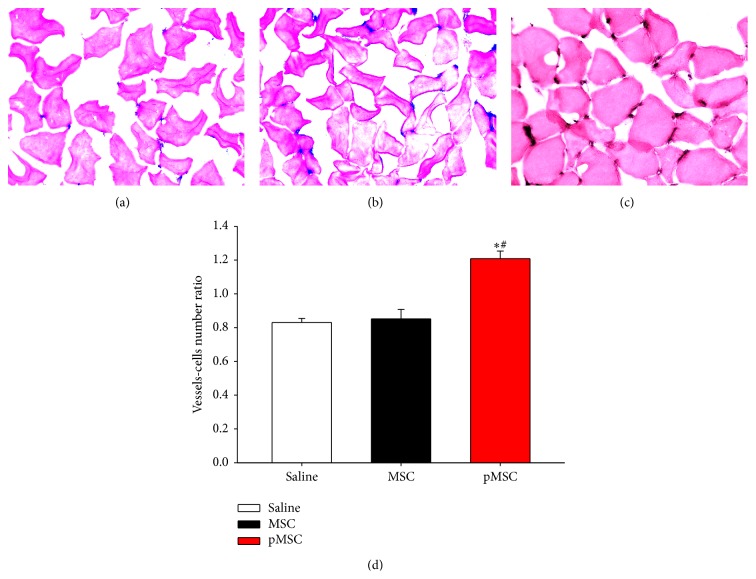
Microvascular density. Blue staining indicates capillaries. Photomicrographs obtained from animals from the saline (a), MSC (b), and pMSC (c) groups. Ratio between vessel number and the number of cells (d). Magnification: 400x. Values indicated as the mean ± sem. ^*∗*^ *p* < 0.05 versus saline; ^#^ *p* < 0.05 versus MSC.

As shown in Figure 6, the treatment with MSCs primed with EGM-2 produced a significant increase in the ratio of blood vessels/number of cells compared to the saline and MSC groups, indicating that these cells were able to induce new vessel formation* in vivo*.

### 3.8. Detection of Transplanted Cells

**Figure 7:**
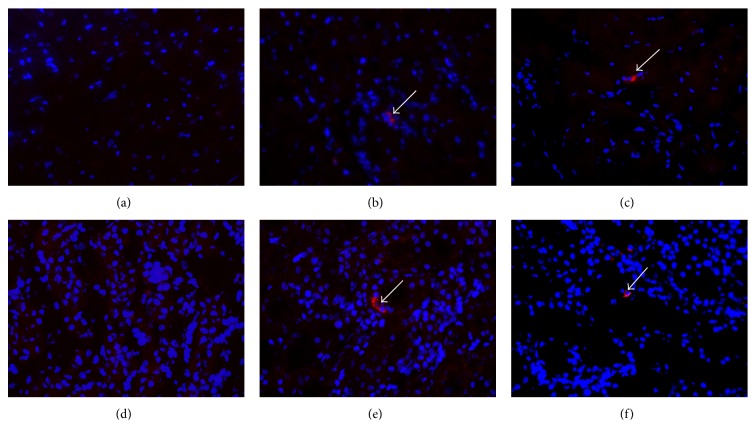
Detection of transplanted cells. Photomicrography of hearts (top panels) and kidneys (bottom panels) from the saline (a, d), MSC (b, e), and pMSC (c, f) groups. DAPI staining in blue and CM-DiI staining in red. Magnification: 400x. White arrows indicate the transplanted cells.

The CM-DiI cell tracker staining of the MSCs before transplantation allowed the detection of cells* in vivo* after 10 days of treatment. Although they were very low in quantity, it was possible to detect the cells of both the MSC and pMSC groups in the heart and kidneys of the animals (Figure 7).

## 4. Discussion

In the present work, we demonstrated the beneficial effects of priming MSCs prior to injection to treat arterial hypertension in an experimental model of spontaneously hypertensive rats. As previously described, we choose SHRs because they are easy to manipulate and have large amounts of available data and the hypertension pathogenesis is quite similar to that in humans.

Priming approach studies have been already evaluated and proven effective in models such as immune modulation, in which priming of MSCs with interferon boosts the MSC immunosuppressive properties [17], and myocardial infarction, in which the beneficial effects of priming MSC to direct them to follow the cardiomyogenic differentiation prior to injection to treat myocardial infarction have been demonstrated [18]. Considering MSC stimulation with EGM-2, König et al. [19] demonstrated that amnion-derived MSCs cultured in EGM-2 for 5–7 days produced an increase in the rate of* in vitro* cellular growth, changed their fibroblast-like cells toward an endothelial-like morphology, and were able to take up acetylated low-density lipoprotein and form endothelial-like networks in the Matrigel assay, although they did not express the mature endothelial cell markers von Willebrand factor and vascular endothelial cadherin. Moreover, stimulating MSCs with epidermal growth factor (EGF) increases cell proliferation and motility and potentiates the capacity of MSCs to secrete vascular endothelial growth factor (VEGF) and hepatocyte growth factor (HGF), which could indicate an improvement in the angiogenic ability of these cells and their therapeutic potential [20]. Studies have also demonstrated that fibroblastic growth factor (FGF) maintains MSCs in an undifferentiated state during proliferation while preserving their multipotentiality. In other words, FGF appears to promote self-renewal and maintain the stemness of MSCs* in vitro* [21].

In the present study, the stimulation of MSC with EGM-2 reduced the basal rate of cell death* in vitro*. These results can be explained by the presence of growth factors in EGM-2 because some of these factors have a marked antiapoptotic effect on MSCs, such as IGF-1 [22], FGF-2 [23], and VEGF [24].

An important finding of this study was the significant long-lasting reduction in the arterial blood pressure levels, approximately 10–20 mmHg, and this effect seems to be related to an improvement in endothelial function. Cellular therapy in the context of cardiovascular diseases is performed, in general, to promote regeneration of injured tissue, for example, tissue injured as a result of a myocardial infarction. Considering that arterial hypertension is the main risk factor associated with cardiovascular events, our approach is preventive rather than therapeutic regarding tissue damage. In this way, the reduction in arterial pressure levels is quite beneficial, although this reduction did not reach normal values. Based on our data, studies have shown benefits promoted by MSC transplantation in the context of monocrotaline-induced pulmonary hypertension, with a reduction in the pulmonary vascular bed resistance, right ventricular systolic pressure, right ventricular weight-body weight ratio, and pulmonary vascular resistance, and these effects seem to be related to improvement in the endothelium-dependent vasodilatation [25, 26]. In the context of renovascular hypertension, weekly transplantation of 2 × 10^5^ MSCs prevented the progressive increase in systolic blood pressure (SBP), improved renal morphology and microvascular rarefaction, and reduced fibrosis, proteinuria, and proinflammatory cytokines [27]. Data from our lab [28, 29] have shown that intravenous infusion of 10^7^ white mononuclear cell extracted from the bone marrow of male SHRs into female SHRs (syngeneic transplantation) decreases arterial pressure (AP) by approximately 15–25 mmHg for two consecutive weeks and improves the vasodilation response to acetylcholine* in vivo*. Although Oh et al. [30] recently described a long-lasting activation of memory T cells and an eventual late rejection of transplanted allogeneic MSCs, the arterial blood pressure reduction observed in our study most likely was not attributed to a casual immunological rejection and consequent activation of a systemic inflammatory response because SHRs are isogenic [28].

The direct measurement of AP (gold standard) was performed 10 days after stem cell transplantation and indicated a long-lasting reduction of AP, which is consistent with other studies [31]. The choice of this period of evaluation occurs because previous data from our lab documented approximately 15 days of an effect using bone marrow mononuclear cells [28, 29]. In a rough correlation, considering the lifespan of SHRs of approximately 73 weeks [32] and the life expectancy of human beings of approximately 70 years [33], we could consider that the therapeutic effect of a single administration of MSC, approximately 1.5 weeks in rats, would be valid for 1.5 years of hypertension in humans. The drugs commonly used to control SAH need to be used daily, which causes interruptions and low treatment compliance, limiting the efficacy of pharmacological therapy for hypertension. Then, the cellular therapeutic modality is interesting because it demonstrates a long-lasting antihypertensive effect.

In an attempt to determinate the mechanisms involved in the antihypertensive roles of MSCs, the present study investigated improvements in parameters known to be deficient in SHRs such as sympathetic autonomic hyperactivity, endothelial dysfunction, and microvascular rarefaction. The modulation of the autonomous nervous system on the cardiovascular system was evaluated through HR and AP variability, and the results indicate that the hypotensive effects of pMSCs do not seem to be related to alterations in sympathetic-vagal balance. On the other hand, the endothelial function was improved in response to treatment with pMSC, which is according to other authors, including Mikami et al. [34]. Similarly, the hypotensive effects of pMSCs were associated with an increase in microvascular density in these animals, indicating an important angiogenic effect of these cells. The angiogenic potential of MSCs is well documented in the literature under various conditions, such as ischemia and tumoral angiogenesis [34–36]. The absence of beneficial effects induced by MSCs cultured in conventional medium on AP, endothelial dysfunction, and microvascular rarefaction are justified by previous data from our lab, which demonstrate a functional impairment of the MSC properties of proliferation and differentiation obtained from SHRs compared with those of Wistar Kyoto rats (unpublished data).

Previous data in the literature describes the distribution of intraperitoneally administered MSCs, which were found to be located in the liver, lung, kidney, and spleen, indicating that these cells can circulate and engraft in different organs [37]. In this study, the transplanted cells were identified in the hearts and kidneys 10 days after therapy. The kidneys play an important role in long-term blood pressure regulation, and the presence of pMSC in these organs may indicate a possible role of these cells in renal function, which is known to be impaired in SAH. The reduction in cardiac hypertrophy observed in the pMSC group is most likely secondary to the reduction in AP, although the detection of cells in this organ may indicate some additional protective effects. Notably, the single observation of transplanted cells in the organs does not necessarily indicate that they play some biological role, but further studies must be conducted to elucidate this question.

The present study has a major limitation regarding the lack of evaluation of angiogenic factors profile after priming MSCs with EGM-2 and also after systemic transplantation in receptor SHRs. In our opinion the priming of MSCs with EGM-2 increases their angiogenic and hence antihypertensive effects via an increase in angiogenic factors secretion. This hypothesis is reinforced by findings reported by Takahashi et al. [38] which have described an increase in angiogenic factors secreted by adipose tissue-derived MSC after seven days in EGM-2 culture. In addition, treatment of MSCs with VEGF [10] or FGF-2 [39], both components of the EGM-2 medium, is able to induce* in vitro* differentiation of MSC towards endothelial-like cells. However, more experiments should be performed to address this issue in the near future.

In conclusion, considering that MSCs constitute effective, easily isolated, safe nontumor forming, and high expansive capacity* in vitro* cells, these cells represent a promising alternative to conventional therapy for arterial hypertension. We demonstrate that it is possible to potentiate the therapeutic efficacy of MSCs prior to treatment, whereas our findings seem to indicate that priming of MSCs with endothelial basal medium boosts stem cell therapy in the treatment of systemic arterial hypertension.

## Figures and Tables

**Table 1:** Effect of stem cell treatment on arterial pressure variability in all three experimental groups.

	Saline (*n* = 10)		MSC (*n* = 11)		pMSC (*n* = 10)	
	Systolic	Diastolic	Systolic	Diastolic	Systolic	Diastolic
Variance (mmHg^2^)	43.3 ± 5.7	38.1 ± 4.7	32.7 ± 5.8	22.8 ± 3.1^*∗*^	27.8 ± 3.7	23.7 ± 3.0^*∗*^
LF (mmHg^2^)	27.8 ± 4.6	24.0 ± 4.1	16.5 ± 2.8	13.0 ± 2.0^*∗*^	18.0 ± 3.1	15.7 ± 2.8
HF (mmHg^2^)	0.9 ± 0.1	1.7 ± 0.3	3.5 ± 0.9	2.7 ± 0.7	0.8 ± 0.1	1.7 ± 0.6

LF = low frequency power spectral density, HF = high frequency power spectral density, and ^*∗*^ *p* < 0.05 versus saline.

**Table 2:** Effect of stem cell treatment on heart rate variability in all three experimental groups.

	Saline (*n* = 10)	MSC (*n* = 11)	pMSC (*n* = 10)
Variance (ms^2^)	24.5 ± 3.8	22.3 ± 4.2	27.7 ± 4.1
LF (ms^2^)	1.7 ± 0.4	0.4 ± 0.1^*∗*^	1.5 ± 0.4
LF (nu)	13.1 ± 2.4	4.3 ± 0.9^*∗*^	8.2 ± 1.9
HF (ms^2^)	7.4 ± 1.2	6.8 ± 1.4	10.7 ± 1.7
HF (nu)	69.6 ± 2.9	71.8 ± 3.1	73.4 ± 2.9
LF/HF ratio	0.2 ± 0.03	0.07 ± 0.02	0.13 ± 0.03

LF = low frequency power spectral density, HF = high frequency power spectral density, nu = normalized units, and ^*∗*^ *p* < 0.05 versus saline.
